# Chronic kidney disease in the global adult HIV-infected population: A systematic review and meta-analysis

**DOI:** 10.1371/journal.pone.0195443

**Published:** 2018-04-16

**Authors:** Udeme E. Ekrikpo, Andre P. Kengne, Aminu K. Bello, Emmanuel E. Effa, Jean Jacques Noubiap, Babatunde L. Salako, Brian L. Rayner, Giuseppe Remuzzi, Ikechi G. Okpechi

**Affiliations:** 1 Division of Nephrology and Hypertension, Groote Schuur Hospital and University of Cape Town, Cape Town, South Africa; 2 Renal Unit, Department of Medicine, University of Uyo, Uyo, Nigeria; 3 Department of Medicine, Groote Schuur Hospital and University of Cape Town, Cape Town, South Africa; 4 Non-Communicable Diseases Research Unit, South African Medical Research Council, Cape Town, Cape Town, South Africa; 5 Division of Nephrology and Immunology, Department of Medicine, University of Alberta, Edmonton, Canada; 6 Renal Unit, Department of Medicine, University of Calabar, Calabar, Nigeria; 7 Department of Medicine, College of Medicine, University of Ibadan, Ibadan, Nigeria; 8 Kidney and Hypertension Research Unit, University of Cape Town, Cape Town, South Africa; 9 IRCCS-Istituto di Ricerche Farmacologiche Mario Negri, Clinical Research Center for Rare Diseases Aldo & Cele Daccò, Bergamo, Italy; Universita degli Studi di Perugia, ITALY

## Abstract

**Introduction:**

The widespread use of antiretroviral therapies (ART) has increased life expectancy in HIV patients, predisposing them to chronic non-communicable diseases including Chronic Kidney Disease (CKD). We performed a systematic review and meta-analysis (PROSPERO registration number CRD42016036246) to determine the global and regional prevalence of CKD in HIV patients.

**Methods:**

We searched PubMed, Web of Science, EBSCO and AJOL for articles published between January 1982 and May 2016. CKD was defined as estimated glomerular filtration rate (eGFR) <60ml/min using the MDRD, Cockcroft-Gault or CKD-EPI equations. Random effects model was used to combine prevalence estimates from across studies after variance stabilization via Freeman–Tukey transformation.

**Result:**

Sixty-one eligible articles (n = 209,078 HIV patients) in 60 countries were selected. The overall CKD prevalence was 6.4% (95%CI 5.2–7.7%) with MDRD, 4.8% (95%CI 2.9–7.1%) with CKD-EPI and 12.3% (95%CI 8.4–16.7%) with Cockcroft–Gault; p = 0.003 for difference across estimators. Sub-group analysis identified differences in prevalence by WHO region with Africa having the highest MDRD-based prevalence at 7.9% (95%CI 5.2–11.1%). Within Africa, the pooled MDRD-based prevalence was highest in West Africa [14.6% (95%CI 9.9–20.0%)] and lowest in Southern Africa (3.2%, 95%CI 3.0–3.4%). The heterogeneity observed could be explained by WHO region, comorbid hypertension and diabetes mellitus, but not by gender, hepatitis B or C coinfection, CD4 count or antiretroviral status.

**Conclusion:**

CKD is common in HIV-infected people, particularly in Africa. HIV treatment programs need to intensify screening for CKD with added need to introduce global guidelines for CKD identification and treatment in HIV positive patients.

## Introduction

Chronic Kidney Disease (CKD) is a worldwide public health problem; moving from 27^th^ to the 18^th^ most important global cause of death within the last 2 decades [1]. This degree of shift was second only to HIV/AIDS [1], suggesting a significant relationship between HIV and CKD as an important intersection between chronic non-communicable diseases (NCDs) and communicable diseases.

With the roll-out of antiretroviral therapies (ARTs), individuals with HIV are now living longer. As a consequence, the spectrum of kidney diseases in HIV patients has broadened, ranging from asymptomatic changes in renal function like proteinuria, [2, 3] electrolyte losses [4] and acute kidney injury [5] occurring from diarrheal illnesses to various degrees of CKD occurring as a result of renal damage from chronic non-communicable diseases or HIV-associated nephropathy (HIVAN). Furthermore, the use of certain medications included in some ART regimens such as tenofovir and ritonavir, has been shown to increase the risk of CKD[6]. Among incident end-stage renal disease (ESRD) patients, HIV has been implicated as the etiologic factor in 0.4%–0.7% of patients in France [7, 8]; 0.5%–1.1% in Spain [9, 10]; 6.6% in Cameroon [11]; and 28.5% in South Africa [12]. One large study has shown that as much as 3.3% of HIV positive patients with normal baseline estimated glomerular filtration rate (eGFR) developed CKD over a relatively short follow up period of 3.7 years, highlighting the burden of kidney disease in HIV patients [13]. The prevalence of CKD in HIV-infected individuals varies widely between geographic regions and depends on the reporting methods and the definition of CKD used, ranging from 2% to 38% [14, 15]. Although there is an increasing number of individual reports on the prevalence of CKD in the HIV population, the data have not been appropriately synthesized to date.

In this analysis, we synthesized available data on CKD prevalence in the adult HIV population at both regional and global levels. The overarching goal was to provide an essential basis to guide contextualized effective prevention and control strategies to tackle the burden of CKD in this population.

## Methods

### Selection of studies for inclusion in the review

**Fig 1:**
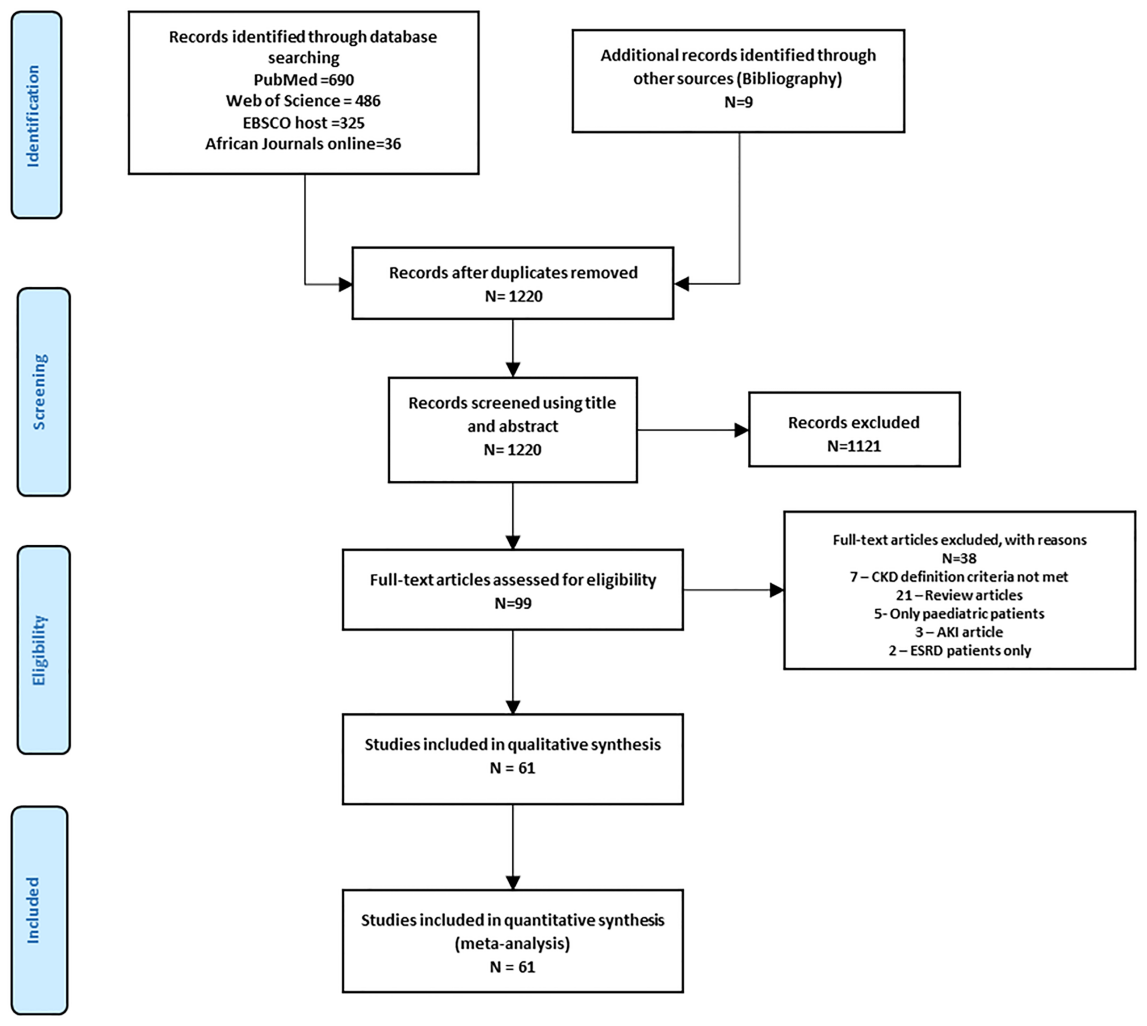
Flow diagram for the selection of studies.

The Preferred Reporting Items for Systematic Reviews and Meta–Analysis (PRISMA) 2009 guidelines [16] served as the template for reporting the present review (S1 Table, Fig 1). The study protocol was published at the International Prospective Register of systematic reviews, (PROSPERO registration number CRD42016036246). All observational studies and clinical trials reporting on the prevalence of CKD in HIV-infected adults (≥ 18 years) or providing enough data to compute it, using established creatinine-based equations [Modification of Diet in Renal Disease (MDRD) [17], Cockcroft–Gault (CG) [18], Chronic Kidney Disease Epidemiology (CKD-EPI) [19]] to estimate GFR were included. CKD was defined as eGFR <60ml/min/1.73m^2^ irrespective of proteinuria status. Studies that reported CKD as eGFR <60ml/min/1.73m^2^ and/or persistent proteinuria were only included if we could compute the frequency of those with eGFR <60 ml/min/1.73m^2^ from available data in the article. We also included studies that reported CKD prevalence using a single estimated eGFR in order to accommodate studies from low-income countries where repeated serum creatinine measurement might not have been performed. A comparison of the pooled prevalence from studies with a single eGFR estimate and that with multiple estimates was also undertaken. We excluded studies with small sample size (<100 participants) and those including both adult and pediatric populations in which it was not possible to disaggregate data for adults. For studies published in more than one report (duplicates), the most comprehensive reporting the largest sample size was considered.

### Identification of studies

We searched PubMed/MEDLINE, EBSCO, Web of Science and African Journals Online to identify all relevant articles reporting data on the prevalence of CKD in HIV-infected adults published from January 1, 1982 (when the HIV epidemic started) to September 30, 2016. We conceived and applied a search strategy based on the combination of relevant terms relating to HIV and CKD. The search strategy for Pubmed, web of science, EBSCO and AJOL is shown in S2 Table. No language restrictions were applied. References of all relevant research articles and reviews were also scrutinized to identify additional potential data sources.

### Assessment of methodological quality of included articles

The methodological quality of included studies was evaluated using the 9-point rating system developed by Stanifer et al [20] and modified for the purposes of this study. The scoring criteria for quality of studies is shown in S3 Table while S4 Table shows the methodological quality of the included articles. The scoring criteria assessed factors related to representativeness of the study participants, sampling, sample size and assessment of possible confounders to the relationship between HIV and CKD. Studies were rated as having a high, medium or low methodological quality when they were assigned a score higher than 6, 5 and 6; or 4 and below respectively.

### Study selection and data extraction

Two investigators (UEE and IGO) independently screened the titles and abstracts of articles retrieved from literature search, and the full-texts of articles found potentially eligible were obtained and further assessed for final inclusion (Fig 1). Disagreements were resolved by consensus or consultation of a third investigator (APK). For clinical trials, we used baseline data. A World Health Organization (WHO) region [21] was assigned to each study depending on the country of recruitment. All studies from Africa were subsequently seperated from the rest and further sub-divided into the different African Union (AU) sub-regions [22] for the purpose of statistical comparisons. Two investigators (UEE and EEE) independently extracted data; discrepancies between investigators were resolved through discussion until consensus was achieved. In one instance [23], an author was contacted for clarification where data was uncertain. Data extracted included first author name, year of publication, country of study origin, WHO region, African sub-region (if study was from Africa), gender proportions in the study population, median age, Body mass index (BMI), CD4 count and viral load of the study population, prevalence of hepatitis B, C co-infection; and the prevalence of hypertension and diabetes mellitus in the study population.

### Statistical analyses

A meta-analysis was used to summarize prevalence data. We pooled the study-specific estimates using a random-effects meta-analysis model (DerSimonian-Laird) to obtain an overall summary estimate of the prevalence of CKD according to the different eGFR equations across studies [24], after stabilizing the variance of individual studies with the use of the Freeman-Tukey double arcsine transformation to minimize the effect of extreme prevalence on the overall estimate [25]. Heterogeneity was assessed using the *χ*^2^ test on Cochrane’s Q statistic [26] and quantified by calculating the I^2^ (with values of 25%, 50% and 75% representing low, medium and high heterogeneity respectively)[27]. Subgroups analysis was also performed using the Q-test based on ANOVA. We assessed the presence of publication bias using funnel plots and the Egger’s test [28]. We assessed inter-rater agreement for study inclusion and data extraction using Cohen’s kappa (κ) coefficient [29]. A p-value <0.05 was considered indicative of statistically significant difference between subgroups. Data was analyzed using the statistical software Open Meta Analyst [30] and the *metaprop* [31] *p*ackage in STATA version 14.0 for Windows (Stata Corp. 2015. Stata Statistical Software: Release 14. College Station, Tx: Stata Corp USA).

## Results

The initial literature search retrieved 1220 articles of which 99 were selected after title and abstract screening for full-text review. Finally, 61 articles [23, 32–91] were eligible and included in this systematic review (Fig 1). There was a high agreement between investigators for study inclusion (κ = 0.81).

Included studies reported on 209,078 HIV-infected adults from 60 countries. There were 46,295 participants (26 studies) from Africa; 52,785 (9 studies) from Europe; 52,305 (11 studies) from North America; 3,661 (4 studies) from South America; 49,147 (9 studies) from Western Pacific and 248 (1 study) from the Eastern Mediterranean. One study [76] from multiple countries in more than two continents had 4,637 HIV–infected adults. MDRD, CG and CKD-EPI equations were used to estimate GFR in 45 studies (n = 167,011 participants), 19 studies (n = 59,414 participants) and 14 studies (n = 41,791 participants) respectively. Thirty-one studies (n = 111,415 participants) used MDRD [32–35, 37–45, 48, 51–54, 57, 59, 62, 65–69, 82, 84, 85, 89] equation only; 7 (n = 16,756 participants) used CKD–EPI [76–80, 83, 86] and 9 (n = 24,622 participants) used CG only [23, 70–75, 81]. Seven articles (n = 31,268 participants) applied MDRD and CG [36, 50, 55, 56, 60, 64, 87]; 4 (n = 20,742 participants) applied MDRD and CKD-EPI [46, 47, 49, 58] while 3 (n = 4,275 participants) applied all 3 equations [61, 63, 88]. Most of the articles were cross-sectional (75.4%); followed by cohort studies (18.0%); then case-control 2 (3.3%); clinical trials 2 (3.3%).

The component studies had a sample size range of 163 [58] to 41,862 [89] participants with the proportion of women ranging from 0% [79] to 100% [88]. The mean age of participants ranged from 31.4 [86] to 48.7 [39] years, and median CD4 count from 147 cells/ul [70] to 651 cells/ul [76]. Some studies [23, 34, 35, 42, 55, 59, 73–77, 80, 89] consisted exclusively of ART–naïve individuals while the rest had varying proportions on ARTs. The prevalence of hepatitis B and C co-infection ranged from 1.6% [33] to 15.1% [70] and from 3.3% [69] to 50.3% [47] respectively. Most of the studies had medium methodological quality (63.9%, n = 39) (S4 Table); 11 studies (18.0%) were of high quality, including 2 (7.7%) studies from Africa, 3 (33.3%) from Europe and 3 (27.3%) from North America. Table 1 provides a summary of data extracted.

**Table 1:** Summary of extracted data from all included studies.

Author	Year	Country	WHO Region	Sample Size	No. of CKD Patients	Age	Male (%)	Female (%)	CD4	VL (log)	ARV naïve (%)	ARV Use (%)	HBV (%)	HCV (%)	HTN (%)	DM (%)	BMI (kg/m^2^)
**MDRD**																	
**Adedeji et al** [32]	2015	Nigeria	AFRO	183	44	37.9	42.6	57.4	201								
**Al-Sheikh et al** [33]	2013	Saudi Arabia	EMRO	248	2	39	66.5	33.5	305	4.8	3	97	1.6	8.6	13.3	16.1	
**Anyabolu et al** [34]	2016	Nigeria	AFRO	375	32	38.8	28	72			100						
**Cao et al** [35]	2013	China	WPRO	538	13	36.5	74.2	25.8	173	4.6	100		14.4	14.9	3.2	3	21.4
**Caihol et al** [36]	2011	Burundi	AFRO	300	5	40.1	29.7	70.3	325	1.65	30.2	69.8	5	5.3	2.7	2	21.8
**Calza et al** [37]	2013	Italy	EURO	894	44	44.2	70.9	29.1	508	2.2	22.5	77.5	5.9	35.9	25.7	6	24.6
**Campbell et al** [38]	2009	UK	EURO	3439	81	42	72.1	27.9	135				3.9	5.1	29.6	22.2	
**Cheung et al** [85]	2007	China	WPRO	322	18	45.2	82	18	50	4.89	6.5	93.5	14.9	4.3	7.4	10.6	
**Choi et al** [39]	2007	USA	AMRO1	12315	1041	48.7	97.7	2.3			86	14			38.9	15.8	
**Cianflone et al** [40]	2010	USA	AMRO1	717	22	41	92	8	515		27	73	5.2	3.9	33	8	
**Colson et al** [41]	2010	Belgium	EURO	2275	68	42.6	70.5	29.5			18.6	81.4					
**Ekat et al** [42]	2012	Congo	AFRO	562	48	38.8	33.9	66.1	192		100						20.3
**Fernando et al** [43]	2008	USA	AMRO1	421	41	43.6	60.2	39.8	422	4.5	11	89	5	32	27.5	2.5	
**Fischer et al** [44]	2010	USA	AMRO1	23155	2833	44	98	2	336	3	83	17	4	40	17	15	
**Flandre et al** [45]	2011	USA	AMRO1	7378	349	31.2	70.3	29.7	365		10.7	89.3	7.4	21.4	16	4	
**Fulop et al** [82]	2010	USA	AMRO1	941	23	40.3	60.7	39.3	335		24.3	75.7	10.2	9.5	30	7	
**George et al** [46]	2011	USA	AMRO1	252	22	49.5	63.5	36.5	375	2.88	50.8	49.2			36.5	11.1	
**Gonzalez et al** [47]	2014	Brazil	AMRO2	195	4	47.6	78.5	21.5	676		7.7	92.3	6.7	50.3	32.6	10.3	
**Gracey et al** [48]	2012	Australia	WPRO	733	45	45.6	93	7			16	84		6	28	5	
**Hsieh et al** [90]	2013	Taiwan	WPRO	512	4	43.2	92	8	206	4.93	45.4	54.5	16	33.4	6.1	0.51	
**Ibrahim et al** [49]	2011	UK	EURO	20132	463	34	78	22	350	3.8	20	80	5.3	7.7			
**Longo et al** [50]	2011	Congo	AFRO	300	9	43	23	77	231		12	88			13		24
**Lucas et al** [51]	2010	Uganda	AFRO	1202	8	30	35.4	64.6									
**Lucas et al** [52]	2008	USA	AFRO	4259	284	38	68	32	180		0		8	50		8	
**Mayor et al** [53]	2010	Puerto Rico	AMRO2	1283	116	40.8	69	31	277	5.7	51.7	48.3		20.1	18.9	9.9	
**Menezes et al** [54]	2011	Brazil	AMRO2	213	18	45.6	51.6	48.4	569			100			20.7	14.1	
**Msango et al** [55]	2013	Tanzania	AFRO	355	61	36.1					100						19.7
**Mulenga et al** [56]	2008	Zambia	AFRO	25779	812	38.5	39.8	60.2	144			100					
**Nakamura et al** [57]	2008	Japan	WPRO	748	121	44.9											
**Obirikorang et al** [58]	2014	Ghana	AFRO	163	16	39.9	22.1	77.9	523		31.9	68.1					
**Okafor et al** [59]	2011	Nigeria	AFRO	383	121	36			239			100					
**Overton et al** [60]	2009	USA	AMRO	845	63	40.3	63.7	36.3	433		63.9	36.1	4.9	12	34.3	6.1	
**Owiredu et al** [61]	2013	Ghana	AFRO	479	46	35.8	28.3	71.1	290		62.4	37.6					
**Peck et al** [62]	2014	Tanzania	AFRO	301	35	38.5	32.2	67.8	297		50	50			16.9	0.7	
**Sarfo et al** [63]	2013	Ghana	AFRO	3137	429	38	33	67	133		39.5	60.5					20.3
**Stohr et al** [64]	2008	Uganda/Zim	AFRO	3316	102	36.8	35	65	86								21.1
**Sorli et al** [65]	2008	Spain	EURO	854	65						12.5	87.5					
**Umeizudike et al** [66]	2012	Nigeria	AFRO	402	38	35	37.8	62.2	223	5.4							22.1
**Wools-Kaloustian** [87]	2007	Kenya	AFRO	373	7	35	32.1	67.9	391								
**Wyatt et al** [67]	2007	USA	AMRO1	1239	73	47.1	57	43	397		16	84	6.3	41			
**Wyatt et al** [88]	2011	Rwanda	AFRO	659	97	34		100	256						4.8	0.5	20.9
**Yanigasawa et al** [69]	2011	Japan	WPRO	732	71	46.7	93.9	6.1	416	1.98	9.3	90.7	7.1	3.3	30.3	7.9	
**Yanigasawa et al** [68]	2014	Japan	WPRO	1447	96	44.4	93.3	6.7	487								
**Zhao et al** [89]	2015	China	WPRO	41862	1377	38	68.5	31.5	220		100			11			
**Muramatsu et al** [84]	2013	Japan	WPRO	1482	99	44.2	93.4	6.6	487								
**CG**																	
**Agbaji et al** [70]	2011	Nigeria	AFRO	491	117	38.8	40.1	59.9	147		100		15.1	11.9			
**Brennan et al** [71]	2011	South Africa	AFRO	890	46	37.1	26.5	73.5	245		21.3	78.7					
**Caihol et al** [36]	2011	Burundi	AFRO	300	15	40.1	29.7	70.3	325	5.3	30.2	69.8	5	5.3	2.7	2	
**Kamkuemah et al** [72]	2015	South Africa	AFRO	1092	18	34	38	62			100						
**Longo et al** [50]	2011	Congo	AFRO	300	30	43	23	77	397		12	88					
**Mizushima et al** [91]	2013	Vietnam	WPRO	771	74	36.4	61.6	38.4	349	1.79	65.2	34.8				4.2	
**Msango et al** [55]	2013	Tanzania	AFRO	355	89	36.1											
**Mulenga et al** [56]	2008	Zambia	AFRO	25779	2240	38.5	39.8	60.2	144		100					19.7	
**Onodugo et al** [73]	2014	Nigeria	AFRO	300	73	38.1	34.7	65.3	273	5.46	100						21.8
**Overton et al** [60]	2009	USA	AMRO1	845	63	39.8	64	36	37	12	37	63	4.9	12	34.3	6.1	
**Owiredu et al** [61]	2013	Ghana	AFRO	479	48	35.2	24	76	57.6	42.4							
**Reid et al** [74]	2007	Uganda/Zim	AFRO	3316	242	37	35	65			100						
**Sakajiki et al** [23]	2014	Nigeria	AFRO	400	64	34	40	60			100						22
**Sarfo et al** [63]	2013	Ghana	AFRO	3137	1186	38	33	67	133		39.5	60.5					20.3
**Schoffelen et al** [81]	2015	Netherland	EURO	16836	460	42.4	83.2	16.8	440		46.6	53.4	6.4	7.6	9.7	3.2	23.4
**Stohr et al** [64]	2008	Uganda	AFRO	3316	242	36.8	35	65	86		100						21.1
**Struik et al** [75]	2011	Malawi	AFRO	526	111	34	33.5	66.5	305		100				6.1	0.6	
**Wools-Kaloustian** [87]	2007	Kenya	AFRO	373	43	35	32.1	67.9	391		100						
**Wyatt et al** [88]	2011	Rwanda	AFRO	659	166	34		100	256		100				4.8	0.5	20.9
**CKD-EPI**																	
**Acchra et al** [76]	2015	START*	-	4637	286	36.8	73.1	26.9	651		100		2.9	3.7	19.2	3.5	
**Bandera et al** [77]	2015	Italy	EURO	7385	206	36	73.4	26.6			100		4.4	24.5	3.1	2.1	
**Bonjoch et al** [78]	2014	Spain	EURO	970	29	48	75.6	24.4	567						19	3	23.8
**Estrella et al** [79]	2011	USA	AMRO1	783	39	47	100		511		27	73			37	13	24.9
**George et al** [46]	2011	USA	AMRO1	252	20	49.5	63.5	36.5	375	2.88	50.8	49.2			36.5	11.1	
**Gonzalez et al** [47]	2014	Brazil	AMRO2	195	3	47.6	78.5	21.5	676		7.7	92.3	6.7	50.3	32.6	10.3	
**Ibrahim et al** [49]	2012	UK	EURO	20132	403	34	78	22	350		20	80	5.3	7.7			
**Obirikorang et al** [58]	2014	Ghana	AFRO	163	6	39.9	22.1	77.9	523		31.9	68.1					
**Odongo et al** [86]	2015	Uganda	AFRO	361	52	31.4	36.3	63.7			100						20
**Owiredu et al** [61]	2013	Ghana	AFRO	479	51	35.8	28.3	71.7	290		62.4	37.6					
**Santiago et al** [83]	2014	Brazil	AMRO2	1970	74	41.6	63.6	36.4	184		17.1	82.9	2.9	6	26.6	9.3	
**Sarfo et al** [63]	2013	Ghana	AFRO	3137	434	38	33	67	133		39.5	60.5					20.3
**Wyatt et al** [88]	2011	Rwanda	AFRO	659	52	34		100	256		100						
**Zachor et al** [80]	2016	South Africa	AFRO	650	15	37.9	34.5	65.5	186		100		13.7		7.8	2.2	24.9

VL = Viral load (in log_10_) BMI = Body Mass index HTN = Hypertension DM = Diabetes Mellitus

*START trials in 35 countries AFRO = African region EURO = Europe EMRO = Eastern Mediterranean WPRO = Western Pacific AMRO1 = North AmericaAMRO2 = South America/Carribean

**Fig 2:**
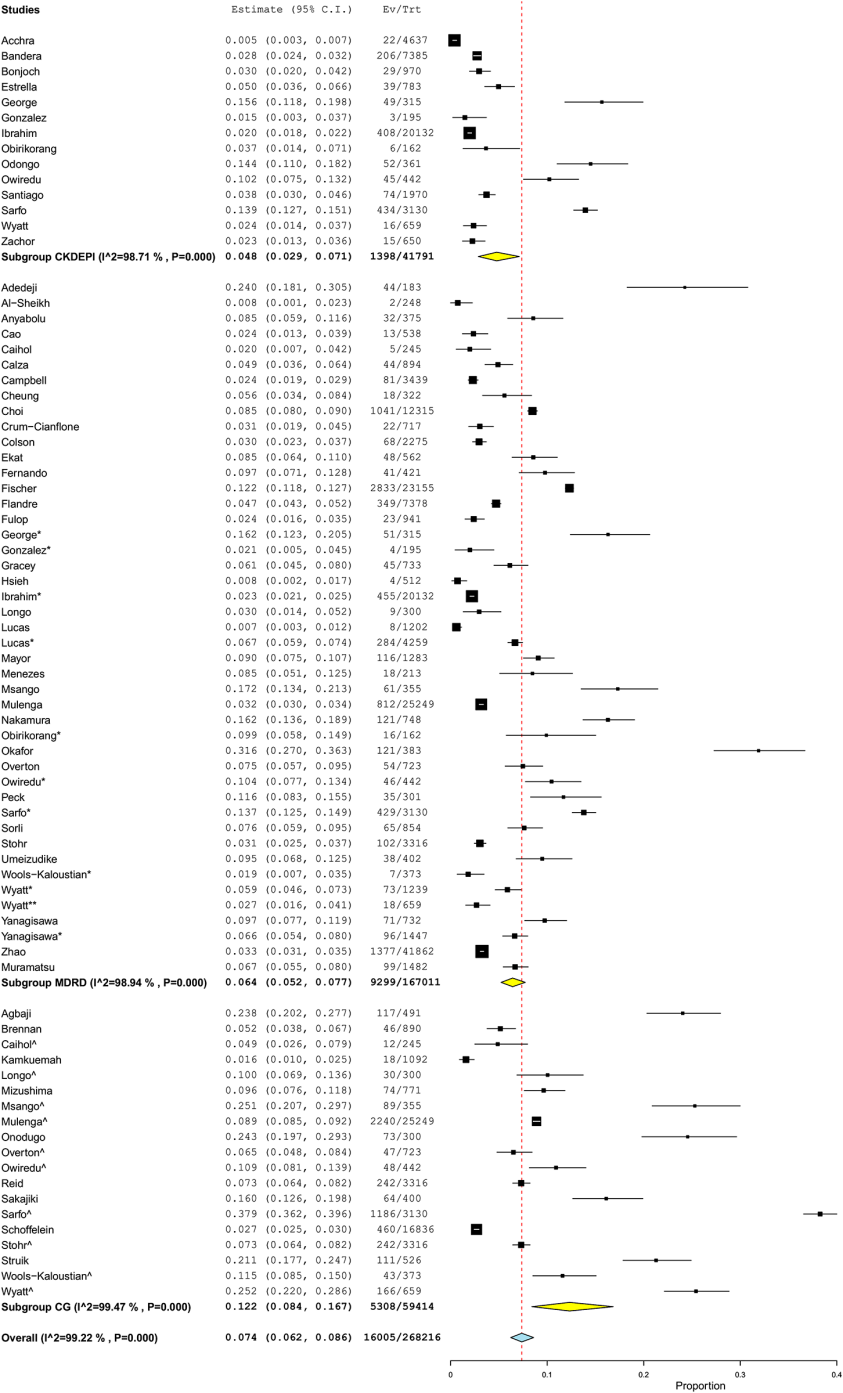
Forest plot showing the overall CKD prevalence in the HIV-infected using the MDRD, CKD-EPI and CG equations. For each study the black box represents the study estimate (prevalence of CKD) and the horizontal bar represents the 95% confidence intervals (95%CI). The yellow diamond at the lower tail for each equation is the pooled effect estimates from random effects models.

**Fig 3:**
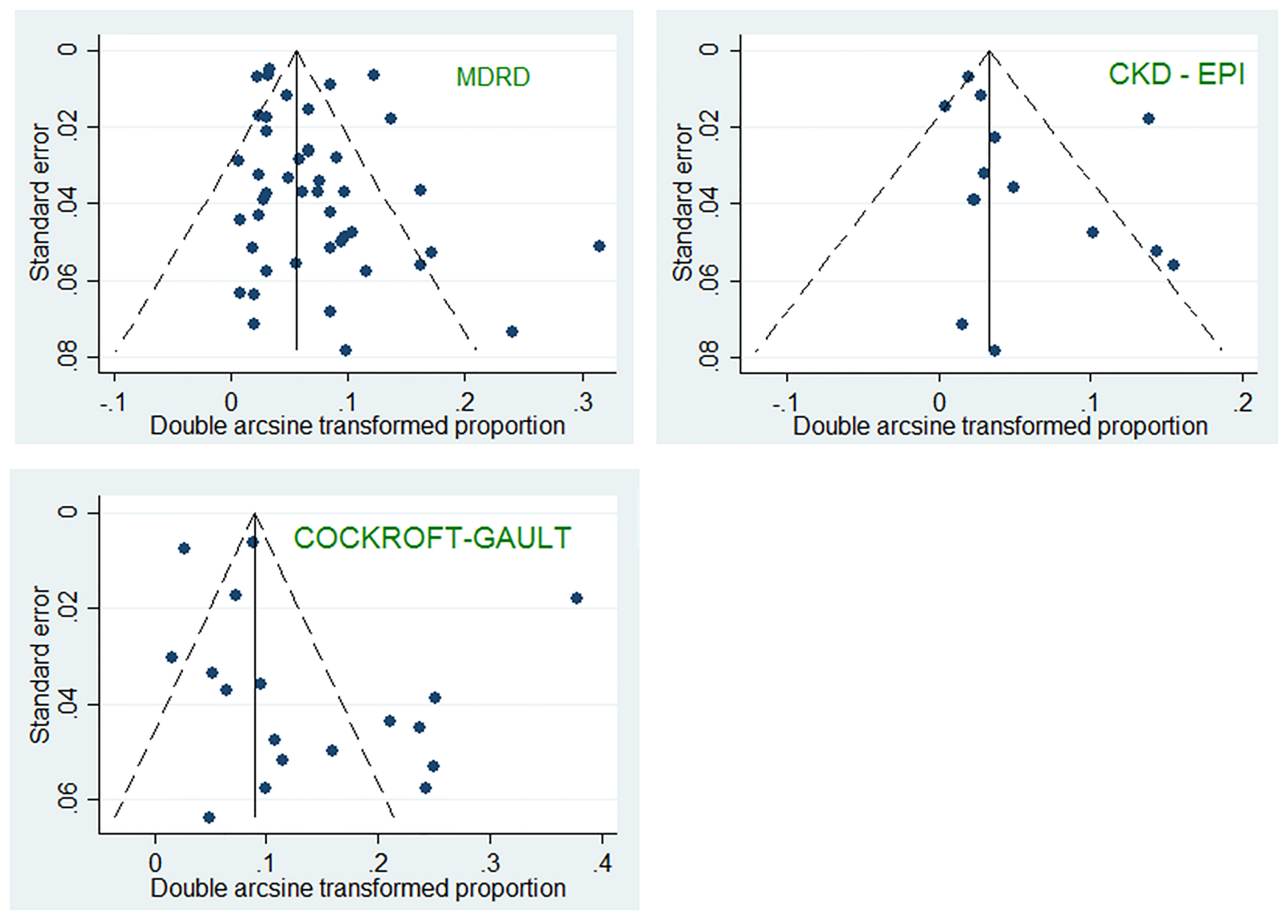
Funnel plots for included studies across different serum creatinine-based GFR equations. For each estimation equation, the arcsine transformed proportion of participants with CKD (relative to the total sample) for each relevant study (horizontal axis) is plotted against its standard error (vertical axis), and represented by the dots. When the dots distribute symmetrically in a funnel shape, this implies an absence of bias. All p-values were >0.05 (Egger test) indicating no evidence of significant publication bias.

The overall prevalence of CKD was 6.4% (95%CI 5.2–7.7%, N = 45 studies, 167,011 participants, I^2^ = 98.9%, heterogeneity-p<0.001) using the MDRD equation, 4.8% (95%CI 2.9–7.1%, N = 14 studies, 41,791 participants, I^2^ = 98.7%; p<0.001) with CKD-EPI and 12.3% (95%CI 8.4–16.7%; N = 19 studies, 59,414 participants, I^2^ = 99.4%, p<0.001) with the CG equation (p = 0.003 for difference across GFR estimators) (Fig 2). There was no evidence of publication bias (Fig 3) all p≥0.147 for the Egger test).

**Fig 4:**
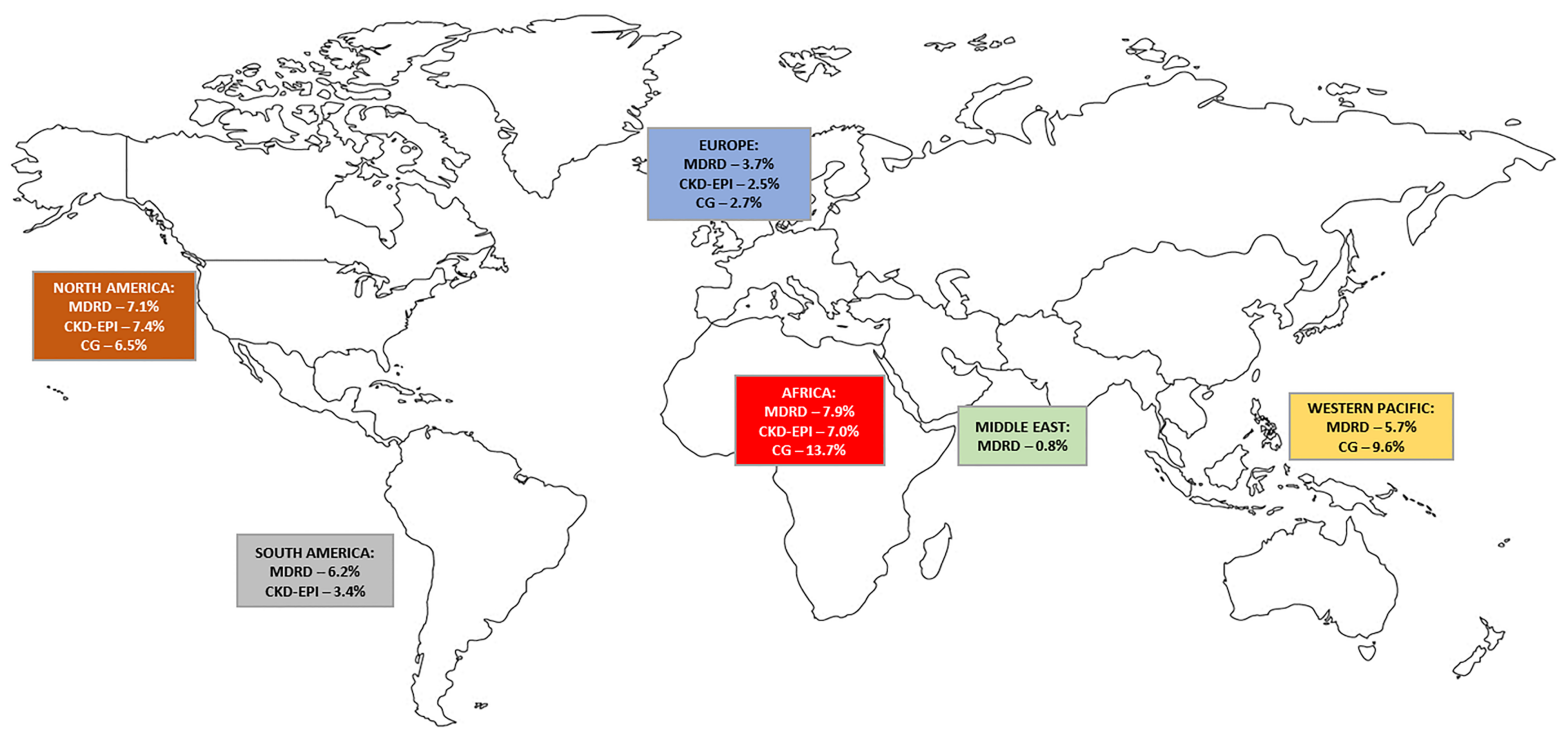
Summary of pooled prevalence of CKD in HIV populations across WHO regions.

Using the MDRD equation, the African region had the highest prevalence estimate at 7.9% (95%CI 5.2%-11.2%) while the European region had the lowest estimate at 3.7% (95%CI 2.5–5.1%); p = 0.004 for difference across regions. Summaries of pooled prevalence by region and GFR estimators are presented in Fig 4 and Table 2; summary statistics from meta-analyses of prevalence studies on CKD in people with HIV using random effects model and arcsine transformations are shown in S5 Table.

**Table 2:** Summary statistics from meta-analyses of prevalence studies on CKD in people with HIV using random effects model and arcsine transformations.

Group	Subgroup	eGFR formula	Number of studies	Number of participants	Number of cases	Prevalence (95%CI)	I^2^ (%)	P—heterogeneity	p-different formulae	p-diff sub-groups	p-Egger test
**WHO Region**	Overall								0.003		
		MDRD	45	167,011	9,299	6.4 (5.2–7.7)	98.9	<0.001		0.004	0.16
		CKD-EPI	14	41,791	1,398	4.8 (2.9–7.1)	98.7	<0.001		<0.001	0.14
		CG	19	59,414	5,308	12.3 (8.4–16.7)	99.4	<0.001		<0.001	0.15
	Africa								0.08		
		MDRD	17	37,639	1,831	7.9 (5.2–11.2)	98.4	<0.001			0.04
		CKD-EPI	6	5,404	568	7.0 (2.8–12.9)	97.5	<0.001			0.21
		CG	16	41,084	4,727	13.7 (9.1–19.0)	99.3	<0.001			0.29
	Europe								<0.001		
		MDRD	5	27,594	713	3.7 (2.5–5.1)	94.6	<0.001			0.09
		CKD-EPI	3	28,487	643	2.5 (1.9–3.2)	87.5	<0.001			0.47
		CG	1	16,836	460	2.7 (2.5–3.0)	-	-			-
	N. America								<0.001		
		MDRD	10	51,463	4,771	7.1 (5.1–9.5)	98.6	<0.001			0.18
		CKD-EPI	2	1,098	88	7.4 (6.0–9.1)	99.8	<0.001			-
		CG	1	723	47	6.5 (4.9–8.5)	-	-			-
	S. America								0.16		
		MDRD	3	1,691	138	6.2 (2.6–11.3)	87.7	<0.001			0.48
		CKD-EPI	2	2,165	77	3.4 (2.7–4.3)	98.2	<0.001			-
		CG	-	-	-	-	-	-			-
	E. Mediterranean										
		MDRD	1	248	2	0.8 (0.2–2.9)	-	-	-	-	-
	W. Pacific										
		MDRD	9	48,376	1,844	5.7 (3.5–8.4)	97.4	<0.001			0.08
		CKD-EPI	-	-	-	-					-
		CG	1	771	74	9.6 (7.7–11.9)	-	-			-
**Africa**											
	Overall								0.09		
		MDRD	17	37,639	1,831	7.9 (5.2–11.2)	98.4	<0.001		<0.001	0.04
		CKD-EPI	6	5,404	568	7.0 (2.8–12.9)	97.5	<0.001		<0.001	0.21
		CG	16	41,084	4,727	13.7 (9.1–19.0)	99.2	<0.001		<0.001	0.29
	West Africa								0.08		
		MDRD	7	5,055	726	14.6 (9.9–20.0)	94.5	<0.001			0.72
		CKD-EPI	3	3,734	485	9.2 (4.8–14.8)	91.5	<0.001			0.09
		CG	5	4,763	1,488	22.0 (11.8–34.3)	98.4	<0.001			0.03
	Southern Africa								<0.001		
		MDRD	2	28,565	914	3.2 (3.0–3.4)	99.9	<0.001			-
		CKD-EPI	1	650	15	2.3 (1.4–3.8)	-	-			-
		CG	6	34,389	2,899	7.6 (5.2–10.4)	97.7	<0.001			0.77
	East Africa								<0.001		
		MDRD	5	2,890	129	5.3 (1.1–12.2)	97.6	<0.001			0.14
		CKD-EPI	2	1,020	68	5.6 (4.3–7.1)	97.1	<0.001			-
		CG	3	1,387	298	20.2 (12.0–29.9)	94.2	<0.001			0.65
	Central Africa								0.19		
		MDRD	3	1107	62	4.2 (1.2–9.0)	89.9	<0.001			0.08
		CKD-EPI	-	-	-	-	-	-			-
		CG	2	545	42	7.5 (5.4–9.9)	94.9	<0.001			-

eGFR—estimated glomerular filtration rate; MDRD—Modification of diet in renal disease, CKD-EPI—Chronic kidney disease Epidemiology collaboration; CG—Cockroft-Gault

CKD prevalence was also high in Africa using the CKD-EPI equation: 7.0% (95%CI 2.8–12.9%). Studies using CG equation were mostly from Africa (84% of the studies), precluding sound regional analysis. The pooled prevalence of CKD in Africa from CG estimator was 13.7% (95%CI 9.1–19.0%); Table 2.

**Fig 5:**
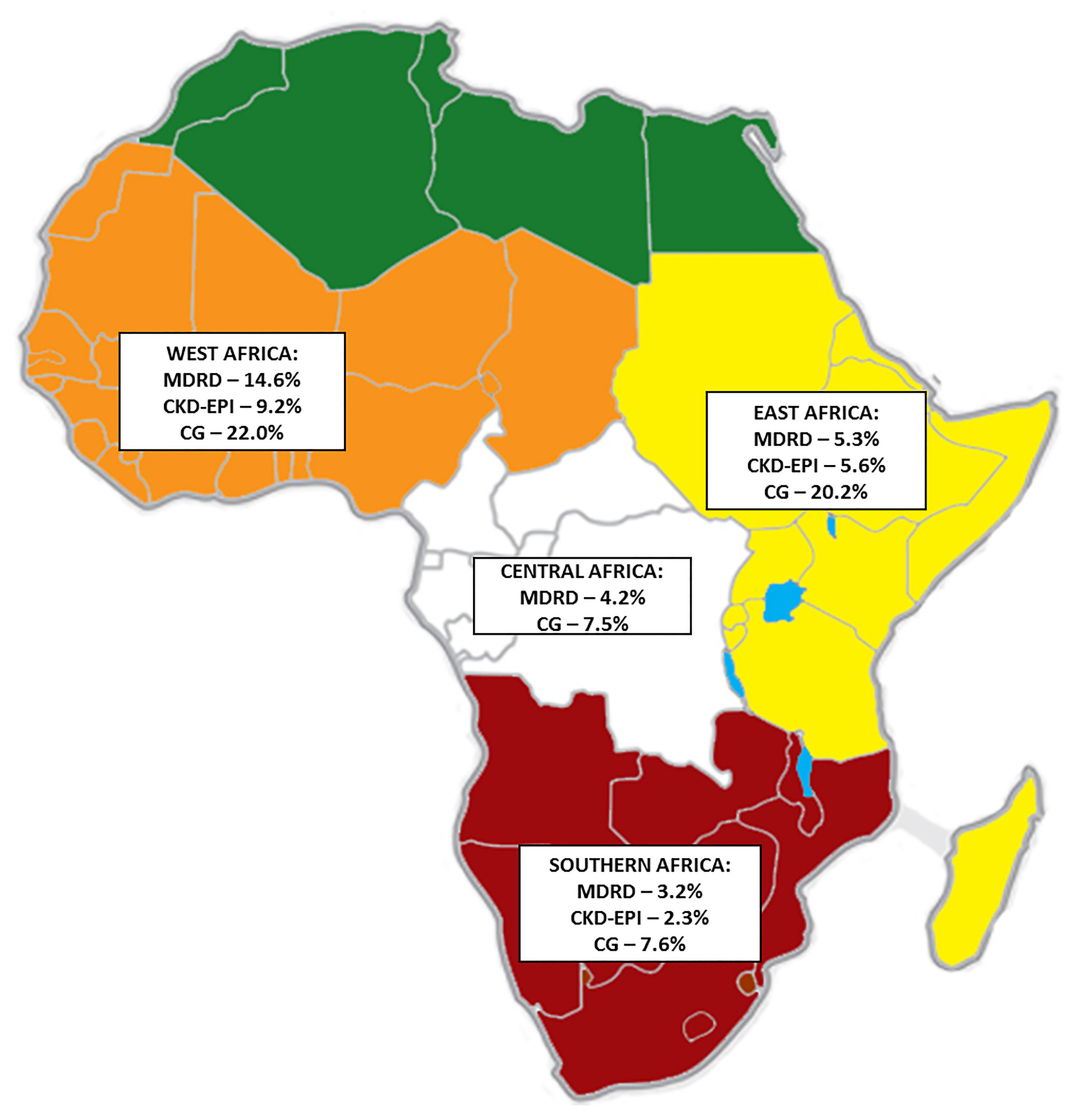
Summary of pooled prevalence of CKD in HIV populations of the African sub-regions.

Of the studies performed in Africa, studies originating from West Africa had the highest pooled prevalence estimate using the MDRD equation: 14.6% (95%CI 9.9–20.0%) while the estimates from Southern Africa (3.2%, 95%CI 3.0–3.4%) were the lowest; p<0.001 for difference across African sub-regions, (Fig 5, Table 2). With the CG equation, West Africa still had the highest estimate, 22.0% (95%CI 11.8–34.3%); East Africa’s estimate was 20.2% (95% CI 12.0–29.9) while Southern Africa had 7.5% (95%CI 5.4–9.9%) (Table 1); p<0.001 for difference across the regions.

One study [88] reported CKD prevalence for only women and another [79] for only men; the pooled prevalence for men compared to women (MDRD) was 4.9% (95%CI 3.1–7.0%) versus 4.5% (95%CI 3.3–5.8%), p = 0.93 for difference by gender. The pooled prevalence (CG equation) for men was 8.3% (95%CI 1.1–20.8%) while that for women was 15.2% (95%CI 4.9–29.7%); p-value = 0.41 for difference by gender, (S5 Table).

Older (≥median age 38.5 years) compared with younger participants (<38.5 years) had lower but non-significant difference in CKD prevalence: MDRD: 6.1% (95%CI 4.6–7.9%) vs. 6.9% (95%CI 5.2–8.8%), p = 0.54; CKD-EPI: 4.8% (95%CI 2.6–7.5%) vs. 4.8% (95%CI 2.3–8.2%), p = 0.98; CG: 8.5% (95%CI 4.5–13.6%) vs. 14.2% (95%CI 7.9–21.9%), p = 0.17. Substantial heterogeneity was apparent within age-groups regardless of the criteria (all p-heterogeneity <0.001). The CKD prevalence rates for patients with co-infection with hepatitis B and C, by level of CD4 count, by ART status and for those with comorbid systemic hypertension and diabetes mellitus are summarized in S5 Table. Importantly, we found that co-infection with hepatitis B or C, level of CD4 count and use of ART did not have a significant effect on CKD prevalence. However, CKD prevalence was significantly increased with comorbid hypertension (MDRD: 20.7% [95%CI 14.3–27.8%] vs 5.4% [95%CI 3.4–7.9%]; p<0.001) or diabetes mellitus (MDRD: 19.4% [95%CI 13.5–26.0%] vs 8.4% [95%CI 5.5–11.8%]; p<0.001) (S5 Table).

Twenty-one studies [35–38, 41, 42, 45, 47, 52, 54, 60, 64, 65, 68, 69, 72, 77, 81, 85, 90, 91] had serum creatinine measured at least twice (four from sub-Saharan Africa [SSA]), a minimum of three months apart. The pooled prevalence for MDRD equation-based studies was 4.7% (95%CI 3.7–5.9%); CKD-EPI 2.6% (95%CI 2.3–3.0%) and CG 5.1% (95%CI 2.8–8.0%), with a significant difference across estimators (p<0.001). The forest plot showing the pooled prevalence of CKD in HIV populations for studies with at least two eGFR estimates using MDRD, CKD-EPI and CG equations is given in S1 Fig. There was no evidence of publication bias as shown in the funnel plots for studies with two or more eGFR values (S2 Fig), p-value = 0.07 for the Egger test).

Using the MDRD equation, the pooled prevalence of CKD reported from African studies that used two eGFR measurements was 4.2% (95%CI 1.4–8.3%) versus 8.9% (95%CI 5.3–13.3) (p-value 0.09) for those that used one eGFR measurement. For studies from North America, the pooled CKD prevalence for studies with at least 2 eGFR measures was 6.1% (95%CI 4.5–7.9%) compared to the pooled prevalence of studies with one eGFR measure of 7.6% (95%CI 5.2–10.4%), p = 0.34. The forest plot showing pooled prevalence for studies with two or more MDRD-based eGFR estimates across the WHO regions is shown in S3 Fig.

## Discussion

To our knowledge, this is the first attempt to provide prevalence estimates for CKD in HIV populations across various WHO regions. Prevalence was highest in Africa and lowest in Europe although the data shows substantial heterogeneity. Despite this, sociodemographic and clinical factors such as gender, age, coinfections with HBV and HCV did not significantly affect the estimates while coincident hypertension and diabetes mellitus had significant effect on the estimates. Paradoxically, our analysis did not reveal significant contribution to CKD prevalence of HIV related factors such as CD4 counts and ART status.

The overall prevalence of CKD in HIV populations is high, regardless of estimator used. This is more so in Africa where the prevalence of CKD in the general population is already high [20]. The high CKD prevalence in HIV patients presents an enormous challenge to health care systems in low to middle income countries (LMICs) with high prevalence of HIV and where access to CKD care is significantly lacking [92]. The clinical and economic implication of a high CKD burden has effects on the functioning of health systems. In higher income countries, high CKD burden may represent remarkable increase in healthcare costs for managing HIV related CKD whereas in LMICs, it may mean enormous pressure on an already weakened and poorly funded health system. The interplay between HIV and CKD also presents an opportunity for integration of chronic non-communicable disease care with communicable disease treatment as this may enhance more effective use of health resources and improve long term outcomes for HIV patients. It is important to determine if there is a higher CKD prevalence among HIV populations than the general population. In Africa, the prevalence of individuals with eGFR less than 60ml/min/1.73m^2^ in the general population is not clear but Stanifer et al[20] reported a pooled prevalence of 13.9% using both eGFR and proteinuria in the definition of CKD. Studies in Sub-saharan Africa have reported a prevalence of eGFR less than 60ml/min/1.73m^2^ of 1.6%[93] to 8.0%[94] using the MDRD formula. In this analysis, the pooled prevalence (using MDRD) was 7.9% for Sub-Saharan Africa. However, studies undertaking head-to-head comparison of CKD prevalence in the HIV–infected population and the general population [95, 96] in climes with better data collection suggest higher CKD prevalence in the HIV population than the general population.

Consistently, there was significant difference in the prevalence reported across the three estimators. Prevalence estimates obtained using the CG equation were generally higher than those obtained from MDRD and CKD-EPI with CKD-EPI being the most conservative of the three. In the general population, the CKD-EPI equation appears to outperform the MDRD and CG equations [97–99]; however, the best equation for GFR estimation and cut-off for definition of CKD in HIV patients remains controversial [100]. Some authors have suggested that existing equations do not take into account the lean muscle mass of malnourished HIV patients and the lipodystrophy associated with ART use [101]. One study report suggests that the CKD-EPI equation may underestimate CKD prevalence in the HIV population in Africans [102]. Whether this is also applicable to European or North American HIV populations, is uncertain. Other studies have supported the idea that eGFR values obtained from CG do not have clinically significant difference from those obtained from CKD-EPI equation in HIV patients and so could be used interchangeably [103], while MDRD is thought to be less sensitive to moderate GFR reductions and thus not useful in HIV patients with early CKD [64]. Noteworthy is the observation that most of the studies from Africa (where patients present with advanced HIV disease) used the Cockroft-Gault equation to estimate GFR either alone or in combination with other creatinine-based formulae. This may be responsible for the relatively higher CKD prevalence obtained from the Cockroft-Gault equation. There have been attempts at validating these creatinine-based estimators in the HIV population [104–106] but there is yet no consensus on the best creatinine-based GFR estimator in this special population.

Although not statistically significant in most of the comparisons, there was clearly a trend towards lower CKD prevalence estimates in the studies with more than one GFR estimate compared with those with only one estimate. This validates the KDIGO position of demonstration of GFR <60ml/min/1.73m^2^ for at least 3 months [107] before a firm diagnosis of CKD is made. This may provide evidence of significant risk of overestimation of CKD prevalence in single eGFR studies because of the possibility of undiagnosed acute kidney injury (AKI) especially in patients with HIV who tend to have higher risk of AKI than the general population [108].

Hypertension and diabetes mellitus remains significant risk factors for CKD in the HIV population as seen in this analysis when head-to-head comparison was performed between HIV only cohorts and HIV/hypertension or DM co-morbidities. Both hypertension and diabetes mellitus are age-related conditions and with the increasing age of HIV patients, a higher prevalence of CKD might be predicted in future in HIV positive patients. Both conditions, however, did not appear to explain some of the heterogeneities in CKD prevalence estimates, when comparison was made based on median hypertension or DM prevalence (S5 Table). This may not be unconnected with the lack of uniformity in the definition or method of assessment of these factors among the constituent studies. For example, one study [38] defined hypertension as blood pressure of at least 160/90mmHg while others [33, 43] used a cut-off of 140/90mmHg. Also, some studies [35, 44, 85] did not provide definition of hypertension while others [36] used patient-reported history of hypertension. Similarly diabetes mellitus had varying definitions ranging from self-reported history of diabetes mellitus [36] to a combination of fasting plasma glucose, random plasma glucose, related symptoms and current use of antidiabetic medication [37, 90] or inadequate information about criteria for diagnosis [82]. However, multivariate regression in some of the component studies [38, 82, 109] identified significant association between diabetes and hypertension with CKD in HIV patients.

The effect of hepatitis B and/or hepatitis C on CKD occurrence in HIV patients has not been consistent. In this study, we found no significant difference in the pooled prevalence of studies with high hepatitis B or C co-infection compared with those with low prevalence of these viral co-infections. Some observational studies have found a higher risk of CKD [35, 90, 110] among hepatitis B or C co-infected HIV patients while others found no significant effect with hepatitis B or C co-infection [36, 37]. A meta-analysis investigating the effect of hepatitis C co-infection on CKD occurrence and progression in HIV patients [111] found significantly increased risk of CKD, proteinuria and AKI in co-infected individuals compared to those with only HIV infection. We are unaware of any published meta-analysis comparing CKD prevalence or progression in HIV-hepatitis B co-infected individuals with HIV only patients though observational studies [110, 112] suggest increased CKD risk with hepatitis B co-infection. Aggregation of data from high-income countries (high HCV co-infection and relatively low CKD prevalence) with LMIC (low HCV co-infection and high CKD-HIV prevalence) may have led to a loss of significant difference in CKD prevalence in the HIV-HCV co-infected compared to those without the co-infection.

One possible reason for the relatively high prevalence of CKD in African patients is late presentation to HIV care clinics at advanced stages of disease. This is evidenced by the significantly lower CD4 counts in African patients compared to the other regions. This may be compounded by late initiation of anti-retroviral medications giving adequate time for HIV–induced or related damage to the kidneys. In North America, ARTs are given to all HIV–infected individuals regardless of CD4 count to reduce morbidity and mortality associated with HIV infection [113]. This is has not been the case in most SSA countries where cut-offs of CD4 counts were used for initiation of ART [South Africa (2013), < 350cells/μl [114]; South Africa (2015), <500 cells/μl [115]; Nigeria (2007), <200/μl [116]; Nigeria (2010), <350 cells/μl [117]]. It was only in 2016 that ART initiation was done regardless of CD4 count in some SSA countries. The effect of this policy change on CKD prevalence among HIV patients may only become apparent in the future. Early initiation of ARTs, especially in blacks, has been proposed as one of the measures for preventing CKD progression among HIV patients [118]. As more patients in SSA access ARTs it is possible that the incidence of CKD may not be too different between individuals of SSA origin compared with Caucasians [81]. There is also the problem of poor and inadequate facilities for long term monitoring of HIV patients on ARTs in Africa which makes early diagnosis of CKD difficult.

Furthermore, CKD in HIV patients may occur because of repeated episodes of undocumented AKI. AKI is common among HIV patients and is an important cause of morbidity and mortality in this patient group with sepsis and hypovolemia from diarrhea being the commonest causes [119–121]. AKI has also been documented as an independent risk factor for future ESRD with increasing ESRD risk associated with worsening AKI stage in HIV patients [109, 122].

The higher prevalence of HIV–related kidney disease in African Americans compared to Caucasian Americans [52] and very high CKD prevalence among HIV patients in West Africa suggests a possible genetic role in the increased CKD prevalence in SSA. This hypothesis is further strengthened by the observation that most African Americans are of West African origin and this study has shown the highest prevalence of CKD in HIV among West Africans. APOL1 and MYH9 polymorphisms have been implicated in conferring possible increased risk of CKD in Africans [123–127] but there may be more, yet to be identified, genetic risk factors. There also may be confounding environmental factors in Africa contributing to the increased CKD risk among HIV patients.

The global HIV population is quite heterogeneous; male preponderance in North America and Europe whereas females constitute 60–70% of the HIV patients in the African studies reviewed. The influence, if any, of gender difference on the CKD prevalence remains unclear. The prevalence of traditional risk factors for CKD like hypertension, diabetes mellitus and Hepatitis C is also higher in North America and Europe than in Africa. The high prevalence of these modifiable CKD risk factors present a window of opportunity for sustaining therapies that may ultimately slow down CKD progression. The experience garnered from chronic care management of HIV could be leveraged as a platform for integration of non-communicable disease services into HIV populations. The different dimensions of HIV care–prevention, diagnosis, enrollment into care, disease management and palliative care—could also be useful for NCDs. The integrated care model appears to have achieved good results in parts of SSA [128] and emphasis on CKD preventive services among the HIV population may reduce the burden of CKD in LMICs.

There is still inadequate information about the best creatinine–based eGFR formula for Africa in general [93] and the HIV population specifically and as our study has not been able to address this, it is a limitation. Some have suggested that non-inclusion of race to the MDRD equation may improve eGFR estimation in Africans [129] but this has not been validated in the HIV population. The use of Cystatin C is not yet widespread in Africa and may not be sustainable in Africa because of the cost. It is important to determine the best measure of CKD in this special population. We did not include individuals with eGFR greater than 60mls/min/1.73m^2^ and persistent proteinuria in this study. If the definition of CKD was made to include persistent proteinuria, then the prevalence of CKD among HIV patients may be much higher than reported in this study. The lack of information on specific antiretroviral drugs and their potential contribution to the burden of CKD in this work is a limitation.

The burden of CKD in HIV positive patients is high globally, particularly in African patients. HIV treatment programs need to intensify routine screening for CKD at baseline and ART follow up clinics using relatively cheap and simple test for urinary proteins. There is now a great need to produce global guidelines for CKD identification and treatment in HIV patients and integrate treatment for chronic non-communicable disease with HIV patient care.

## Supporting information

S1 Table PRISMA 2009 checklist. (DOCX)

S2 Table Search strategy for Pubmed, web of science, EBSCO host and AJOL. (DOCX)

S3 Table Scoring criteria for quality of studies. (DOCX)

S4 Table Assessment of methodological quality of included articles. (DOCX)

S5 Table Summary statistics from meta-analyses of prevalence studies on CKD in people with HIV random effects model and arcsine transformations (subgroup analyses of gender, ARV status, CD4 count levels, Age groups, and co-morbid hypertension, diabetes mellitus, hepatitis B and C infection). (DOCX)

S1 Fig Forest plot showing the pooled prevalence of CKD in HIV populations for studies with at least two eGFR estimates using MDRD, CKD-EPI and CG equations. (TIF)

S2 Fig Funnel plots for studies with two or more eGFR values. (TIF)

S3 Fig Forest plot showing pooled prevalence for studies with two or more MDRD-based eGFR estimates across the WHO regions. (TIF)
