# Supplementary material for: Chronic kidney disease in the global adult HIV-infected population: A systematic review and meta-analysis
Source: PLoS One. 2018 Apr 16;13(4):e0195443. doi: 10.1371/journal.pone.0195443 (PMC5901989; doi:10.1371/journal.pone.0195443)
Supplement: S2 Table — (DOCX) [file pone.0195443.s002.docx]

**S2 Table**

**Pubmed**

| Search | Add to Builder | Query |
| --- | --- | --- |
| #1 |  | Search ((((((Chronic kidney disease) OR CKD) OR Chronic renal insufficiency) OR Chronic kidney failure) OR Chronic renal failure) OR Chronic kidney insufficiency) OR GFR<60ml/min |
| #2 |  | Search (((HIV) OR Human immunodeficiency virus) OR AIDS) OR Acquired immune deficiency syndrome |
| #3 |  | Search (Prevalence) OR incidence |
| #4 |  | Search ("1982/01/01"[Date - Publication] : "2016/09/30"[Date - Publication]) |

**Web of Science**

| **Set** | Web of Science Core Collection Search History - |
| --- | --- |
| #1 | TS=(Chronic Kidney Disease OR Chronic Renal insufficiency OR Chronic OR chronic Renal failure OR  kidney dysfunction)  *DocType=All document types; Language=All languages;* |
| #2 | TS=(Human Immunodeficiency Virus OR HIV)  *DocType=All document types; Language=All languages;* |
| #3 | #2 AND #1  *DocType=All document types; Language=All languages;* |
| #4 | TS=(Prevalence OR frequency OR incidence)  *DocType=All document types; Language=All languages;* |
| #5 | #4 AND #3  *DocType=All document types; Language=All languages;* |

**EBSCOhost**

S2 ("Chronic Kidney Disease+")

S3 prevalence OR epidemiology OR frequency

S4 HIV OR human immunodeficiency virus

S5 AIDS OR acquired immunodeficiency syndrome

S6 S4 OR S5 ((HIV OR human immunodeficiency virus) OR (AIDS OR acquired immunodeficiency syndrome))

S7 S2 OR Chronic Kidney failure (Chronic Kidney insufficiency OR Renal failure)

S8 S6 AND S7 (((HIV OR human immunodeficiency virus) OR (AIDS OR acquired immunodeficiency syndrome)) AND (Chronic Kidney insufficiency OR Renal failure))

S9 S3 AND S8 (prevalence OR epidemiology OR frequency) AND (((HIV OR human immunodeficiency virus) OR (AIDS OR acquired immunodeficiency syndrome)) AND (Chronic Kidney insufficiency OR Renal failure))

**AJOL**

Chronic kidney disease) OR CKD) OR Chronic renal insufficiency) OR Chronic kidney failure) OR Chronic renal failure) OR Chronic kidney insufficiency) AND renal disease)) AND ((HIV) OR Human Immunodeficiency virus)) AND ((prevalence) OR incidence
