# Supplementary material for: Chronic kidney disease in the global adult HIV-infected population: A systematic review and meta-analysis
Source: PLoS One. 2018 Apr 16;13(4):e0195443. doi: 10.1371/journal.pone.0195443 (PMC5901989; doi:10.1371/journal.pone.0195443)
Supplement: S3 Table — (DOCX) [file pone.0195443.s003.docx]

**S3 Table: SCORING CRITERIA FOR QUALITY OF STUDIES (Adapted from Stanifer et al. Lancet Glob Health 2014; 2 e174 – 181.**

|  | **CRITERIA QUESTION** | **YES(1)** | **NO (0)** |
| --- | --- | --- | --- |
| 1 | Are the study participants representative of the HIV population in the country of study? |  |  |
| 2 | Did the study exclude individuals with other established risk factors for CKD? (These factors may include Hypertension, obesity, underweight and Diabetes) |  |  |
| 3 | Is the sample size adequate (1,000 will be regarded as adequate) |  |  |
| 4 | Were the study participants recruited at random? (Non-probability sampling methods will be considered inadequate) |  |  |
| 5 | Was the response rate at least 60% of the initial sample size? |  |  |
| 6 | Was the exclusion rate less than 10% of the total sample? |  |  |
| 7 | Was eGFR defined as being ≤ 60ml/min/1.73m^2^ using the MDRD/CKD-EPI or Cockroft-Gault formulae? |  |  |
| 8 | Was prevalence reported by ARV status? (A differentiation of prevalence in ARV – naïve patients and those on ARV). |  |  |
| 9 | Were the sociodemographic characteristics of the study participants adequately characterized? |  |  |

**High quality – 7 – 9; Medium quality – 4-6; Low quality – less than 4**
