# Supplementary material for: Chronic kidney disease in the global adult HIV-infected population: A systematic review and meta-analysis
Source: PLoS One. 2018 Apr 16;13(4):e0195443. doi: 10.1371/journal.pone.0195443 (PMC5901989; doi:10.1371/journal.pone.0195443)
Supplement: S4 Table — (DOCX) [file pone.0195443.s004.docx]

**S4 Table: Assessment of methodological quality of included articles**

| **AUTHOR NAME** | **STUDY DESIGN** | **Q1** | **Q2** | **Q3** | **Q4** | **Q5** | **Q6** | **Q7** | **Q8** | **Q9** | **TOTAL** | **QUALITY** |
| --- | --- | --- | --- | --- | --- | --- | --- | --- | --- | --- | --- | --- |
| Adedeji et al | cross-sectional | 0 | 1 | 0 | 0 | 1 | 1 | 1 | 0 | 1 | 5 | MEDIUM |
| Al-Sheikh et al | cross-sectional | 0 | 0 | 0 | 1 | 1 | 0 | 1 | 0 | 1 | 4 | LOW |
| Anyabolu et al | cross-sectional | 1 | 1 | 0 | 1 | 1 | 1 | 1 | 0 | 1 | 7 | MEDIUM |
| Cao et al | cross-sectional | 1 | 0 | 0 | 1 | 1 | 1 | 1 | 0 | 1 | 6 | MEDIUM |
| Caihol et al | cross-sectional | 1 | 0 | 0 | 1 | 1 | 1 | 1 | 0 | 1 | 6 | MEDIUM |
| Calza et al | cross-sectional | 1 | 0 | 0 | 1 | 1 | 1 | 1 | 0 | 1 | 6 | MEDIUM |
| Campbell et al | cross-sectional | 1 | 0 | 1 | 1 | 1 | 1 | 1 | 0 | 0 | 6 | MEDIUM |
| Cheung et al | cross-sectional | 1 | 0 | 0 | 1 | 1 | 1 | 1 | 0 | 1 | 6 | MEDIUM |
| Choi et al | cross-sectional | 1 | 0 | 1 | 1 | 1 | 1 | 1 | 1 | 1 | 8 | HIGH |
| Cianflone et al | cross-sectional | 0 | 0 | 0 | 1 | 1 | 1 | 1 | 1 | 1 | 6 | MEDIUM |
| Colson et al | cross-sectional | 1 | 1 | 0 | 1 | 1 | 1 | 1 | 1 | 1 | 8 | MEDIUM |
| Ekat et al | cross-sectional | 1 | 0 | 0 | 0 | 0 | 1 | 1 | 0 | 1 | 4 | LOW |
| Fernando et al | cross-sectional | 1 | 0 | 0 | 1 | 1 | 0 | 1 | 0 | 1 | 5 | MEDIUM |
| Fischer et al | cross-sectional | 0 | 0 | 1 | 1 | 1 | 1 | 1 | 0 | 1 | 6 | MEDIUM |
| Flandre et al | retrospective cohort | 0 | 0 | 1 | 1 | 1 | 1 | 1 | 0 | 1 | 6 | HIGH |
| George et al | nested case-control | 1 | 0 | 0 | 1 | 1 | 1 | 1 | 0 | 1 | 6 | MEDIUM |
| Gonzalez et al | cross-sectional | 0 | 0 | 0 | 0 | 1 | 1 | 1 | 0 | 0 | 3 | LOW |
| Gracey et al | cross-sectional | 1 | 0 | 0 | 1 | 1 | 1 | 1 | 0 | 1 | 6 | MEDIUM |
| Ibrahim et al | retrospective cohort | 1 | 1 | 1 | 1 | 1 | 1 | 1 | 0 | 1 | 8 | HIGH |
| Longo et al | cross-sectional | 0 | 0 | 0 | 0 | 1 | 1 | 1 | 1 | 1 | 5 | MEDIUM |
| Lucas et al | retrospective cohort | 1 | 0 | 1 | 1 | 1 | 1 | 1 | 0 | 0 | 6 | MEDIUM |
| Lucas et al2 | retrospective cohort | 1 | 1 | 1 | 1 | 1 | 1 | 1 | 0 | 1 | 8 | HIGH |
| Mayor et al | retrospective cohort | 1 | 0 | 1 | 1 | 1 | 1 | 1 | 0 | 1 | 7 | HIGH |
| Menezes et al | cross-sectional | 1 | 0 | 0 | 1 | 1 | 1 | 1 | 0 | 1 | 6 | MEDIUM |
| Mizushima et al | cross-sectional | 1 | 0 | 0 | 1 | 0 | 0 | 1 | 0 | 1 | 4 | MEDIUM |
| Msango et al | cross-sectional | 1 | 0 | 0 | 0 | 1 | 1 | 1 | 1 | 1 | 6 | MEDIUM |
| Mulenga LB | cross-sectional | 1 | 1 | 1 | 1 | 1 | 1 | 1 | 0 | 0 | 7 | HIGH |
| Nakamura et al | retrospective cohort | 1 | 0 | 0 | 1 | 1 | 1 | 1 | 0 | 1 | 6 | MEDIUM |
| Obirikorang et al | case-control | 1 | 0 | 0 | 1 | 0 | 1 | 1 | 0 | 0 | 4 | LOW |
| Odongo et al | cross-sectional | 1 | 1 | 0 | 0 | 0 | 0 | 1 | 0 | 1 | 4 | LOW |
| Okafor et al | cross-sectional | 0 | 1 | 0 | 1 | 1 | 1 | 1 | 0 | 0 | 5 | MEDIUM |
| Overton et al | cross-sectional | 1 | 0 | 0 | 1 | 1 | 0 | 1 | 0 | 1 | 5 | MEDIUM |
| Owiredu et al | cross-sectional | 1 | 1 | 0 | 1 | 1 | 1 | 1 | 1 | 0 | 7 | MEDIUM |
| Peck et al | cross-sectional | 1 | 0 | 0 | 1 | 0 | 0 | 1 | 1 | 1 | 5 | MEDIUM |
| Sarfo et al | retrospective cohort | 1 | 0 | 1 | 1 | 1 | 1 | 1 | 1 | 1 | 8 | HIGH |
| Stohr W et al | Cohort | 1 | 0 | 1 | 1 | 0 | 0 | 1 | 1 | 1 | 6 | MEDIUM |
| Sorli et al | cross-sectional | 1 | 0 | 1 | 1 | 0 | 0 | 1 | 1 | 0 | 5 | MEDIUM |
| Umezudike et al | cross-sectional | 1 | 1 | 0 | 1 | 1 | 1 | 1 | 0 | 0 | 6 | MEDIUM |
| Wools-Kaloustian etal | cross-sectional | 1 | 1 | 0 | 1 | 0 | 0 | 1 | 0 | 1 | 5 | MEDIUM |
| Wyatt et al | cross-sectional | 1 | 0 | 1 | 1 | 0 | 1 | 1 | 0 | 1 | 6 | MEDIUM |
| Wyatt et al (Rwanda) | cross-sectional | 0 | 0 | 0 | 1 | 1 | 1 | 1 | 0 | 1 | 5 | MEDIUM |
| Yanigasawa et al | cross-sectional | 1 | 0 | 0 | 1 | 1 | 1 | 0 | 0 | 1 | 5 | MEDIUM |
| Yanigasawa et al | cross-sectional | 1 | 0 | 1 | 1 | 1 | 1 | 0 | 0 | 1 | 6 | MEDIUM |
| Zhao et al | cross-sectional | 1 | 0 | 1 | 1 | 1 | 0 | 1 | 0 | 1 | 6 | MEDIUM |
| Agbaji et al | cross-sectional | 0 | 0 | 0 | 1 | 0 | 0 | 1 | 0 | 0 | 2 | LOW |
| Brennan et al | retrospective cohort | 0 | 0 | 0 | 1 | 1 | 0 | 1 | 0 | 1 | 4 | LOW |
| Kamkuemah et al | prospective cohort | 0 | 0 | 1 | 0 | 1 | 0 | 1 | 0 | 0 | 3 | LOW |
| Onodugo et al | cross-sectional | 1 | 1 | 0 | 1 | 0 | 0 | 1 | 0 | 0 | 4 | LOW |
| Reid et al | cross-sectional | 0 | 0 | 1 | 1 | 1 | 1 | 1 | 0 | 0 | 5 | MEDIUM |
| Struik et al | cross-sectional | 1 | 0 | 0 | 1 | 1 | 1 | 1 | 0 | 0 | 5 | MEDIUM |
| Acchra et al | prospective cohort | 0 | 0 | 1 | 1 | 1 | 1 | 1 | 0 | 1 | 6 | MEDIUM |
| Bandera et al | prospective cohort | 1 | 0 | 1 | 1 | 1 | 1 | 1 | 1 | 1 | 8 | HIGH |
| Bonjoch et al | cross-sectional | 1 | 0 | 0 | 1 | 1 | 1 | 1 | 0 | 1 | 6 | MEDIUM |
| Estrella et al | Cross-sectional | 1 | 0 | 0 | 0 | 1 | 1 | 1 | 0 | 1 | 5 | MEDIUM |
| Zachor et al | retrospective cohort | 0 | 0 | 0 | 1 | 1 | 1 | 1 | 0 | 1 | 5 | MEDIUM |
| Santiago et al |  | 1 | 0 | 1 | 1 | 1 | 1 | 1 | 1 | 1 | 8 | MEDIUM |
| Sakajiki et al | cross-sectional | 0 | 0 | 0 | 1 | 1 | 1 | 1 | 0 | 0 | 4 | LOW |
| Schoffelen et al | cross-sectional | 1 | 0 | 1 | 1 | 1 | 1 | 1 | 0 | 1 | 7 | HIGH |
| Fulop et al | cross-sectional | 1 | 0 | 0 | 1 | 1 | 1 | 1 | 1 | 1 | 7 | HIGH |
| Hsieh et al | cross-sectional | 1 | 0 | 0 | 1 | 0 | 0 | 1 | 1 | 0 | 4 | LOW |
| Muramatsu et al | cross-sectional | 1 | 0 | 1 | 1 | 1 | 1 | 1 | 0 | 1 | 7 | HIGH |
