# Supplementary material for: Chronic kidney disease in the global adult HIV-infected population: A systematic review and meta-analysis
Source: PLoS One. 2018 Apr 16;13(4):e0195443. doi: 10.1371/journal.pone.0195443 (PMC5901989; doi:10.1371/journal.pone.0195443)
Supplement: S5 Table — (DOCX) [file pone.0195443.s005.docx]

**S5 Table: Summary statistics from meta-analyses of prevalence studies on CKD in people with HIV using random effects model and arcsine transformations**

| **Group** | **Subgroup** | **eGFR formula** | **Number of studies** | **Number of participants** | **Number of cases** | **Prevalence (95%CI)** | **I^2^ (%)** | **P -heterogeneity** | **p-different formulae** | **p-diff sub-groups** | **p-Egger test** |
| --- | --- | --- | --- | --- | --- | --- | --- | --- | --- | --- | --- |
| **Gender** |  |  |  |  |  |  |  |  |  |  |  |
|  | Overall |  |  |  |  |  |  |  | 0.007 |  |  |
|  |  | MDRD | 19 | 147,637 | 6,612 | 4.7 (3.6 – 6.0) | 97.6 | <0.001 |  | 0.93 | 0.48 |
|  |  | CKD-EPI | 3 | 4,599 | 164 | 3.3 (2.5-4.3) | 32.7 | <0.001 |  | <0.001 | 0.36 |
|  |  | CG | 9 | 6,205 | 674 | 12.0 (5.0-21.3) | 98.1 | <0.001 |  | 0.41 | 0.44 |
|  | Male |  |  |  |  |  |  |  | <0.001 |  |  |
|  |  | MDRD | 9 | 73,489 | 3,297 | 4.9 (3.1-7.0) | 98.8 | <0.001 |  |  | 0.55 |
|  |  | CKD-EPI | 1 | 1,970 | 74 | 3.9 (3.0-5.1) | - | <0.001 |  |  | - |
|  |  | CG | 4 | 2,773 | 254 | 8.3 (1.1-20.8) | 96.6 | <0.001 |  |  | 0.12 |
|  | Female |  |  |  |  |  |  |  | 0.005 |  |  |
|  |  | MDRD | 10 | 74,148 | 3,315 | 4.5 (3.3-5.8) | 85.9 | <0.001 |  |  | 0.72 |
|  |  | CKD-EPI | 2 | 2,629 | 90 | 3.0 (2.1-3.9) | 98.4 | <0.001 |  |  | - |
|  |  | CG | 5 | 3,432 | 420 | 15.2 (4.9-29.7) | 98.7 | <0.001 |  |  | 0.34 |
| **ARV status** |  |  |  |  |  |  |  |  |  |  |  |
|  | Overall |  |  |  |  |  |  |  | <0.001 |  |  |
|  |  | MDRD | 21 | 105,846 | 3,688 | 5.4 (4.5-6.4) | 96.8 | <0.001 |  | 0.19 | 0.02 |
|  |  | CKD-EPI | 7 | 35,330 | 780 | 3.0 (1.8-4.5) | 97.4 | <0.001 |  | 0.52 | 0.30 |
|  |  | CG | 8 | 32,073 | 2,824 | 10.4 (7.3-14.1) | 97.6 | <0.001 |  | 0.27 | 0.48 |
|  | ARV - naive |  |  |  |  |  |  |  | 0.06 |  |  |
|  |  | MDRD | 5 | 43,720 | 1,591 | 9.0 (3.0-17.7) | 98.7 | <0.001 |  |  | 0.22 |
|  |  | CKD-EPI | 4 | 13,033 | 295 | 3.6 (1.1-7.4) | 98.5 | <0.001 |  |  | 0.41 |
|  |  | CG | 5 | 5,634 | 508 | 12.4 (5.4-21.8) | 98.5 | <0.001 |  |  | 0.18 |
|  | ARV - exposed |  |  |  |  |  |  |  | 0.002 |  |  |
|  |  | MDRD | 16 | 62,126 | 2,097 | 4.6 (3.7-5.6) | 95.1 | <0.001 |  |  | 0.02 |
|  |  | CKD-EPI | 3 | 22,297 | 485 | 2.5 (1.3-4.0) | 90.1 | <0.001 |  |  | 0.67 |
|  |  | CG | 3 | 26,439 | 2,316 | 7.8 (5.2-10.8) | 89.2 | <0.001 |  |  | 0.65 |
| **CD4 COUNT** |  |  |  |  |  |  |  |  |  |  |  |
|  | Overall |  |  |  |  |  |  |  | 0.007 |  |  |
|  |  | MDRD | 37 | 147,185 | 7,799 | 6.2 (4.9-7.7) | 98.8 | <0.001 |  | 0.37 | 0.22 |
|  |  | CKD-EPI | 12 | 34,045 | 1,140 | 4.4 (2.2 – 7.3) | 98.9 | <0.001 |  | 0.59 | 0.27 |
|  |  | CG | 15 | 54,251 | 4,895 | 12.7 (8.1 – 18.1) | 99.3 | <0.001 |  | 0.33 | 0.19 |
|  | <200 cells/ul |  |  |  |  |  |  |  | 0.09 |  |  |
|  |  | MDRD | 8 | 40,815 | 1,787 | 6.2 (4.9-7.7) | 98.8 | <0.001 |  |  | 0.37 |
|  |  | CKD-EPI | 3 | 5,750 | 523 | 4.4 (2.2-7.3) | 97.1 | <0.001 |  |  | 0.48 |
|  |  | CG | 4 | 32,186 | 3,785 | 17.9 (6.5-33.4) | 98.7 | <0.001 |  |  | 0.44 |
|  | ≥ 200 cells/ul |  |  |  |  |  |  |  | 0.02 |  |  |
|  |  | MDRD | 29 | 107,339 | 2,708 | 6.5 (4.9 – 8.4) | 99.1 | <0.001 |  |  | 0.34 |
|  |  | CKD-EPI | 9 | 28,295 | 617 | 3.9 (2.2-6.1) | 99.1 | <0.001 |  |  | 0.11 |
|  |  | CG | 11 | 22,065 | 1,110 | 11.0 (6.1-17.0) | 99.8 | <0.001 |  |  | 0.007 |
|  | <350 cells/ul |  |  |  |  |  |  |  | 0.02 |  |  |
|  |  | MDRD | 24 | 139,241 | 7,262 | 6.0 (4.4-7.3) | 88.6 | <0.001 |  |  | 0.37 |
|  |  | CKD-EPI | 6 | 26,983 | 992 | 5.0 (1.6-10.1) | 97.5 | <0.001 |  |  | 0.40 |
|  |  | CG | 12 | 36,319 | 4,345 | 14.6 (8.8-21.4) | 97.2 | <0.001 |  |  | 0.31 |
|  | ≥350 cells/ul |  |  |  |  |  |  |  | 0.16 |  |  |
|  |  | MDRD | 13 | 8,913 | 596 | 6.0 (4.4-7.8) | 99.3 | <0.001 |  |  | 0.51 |
|  |  | CKD-EPI | 6 | 7,062 | 148 | 3.9 (1.0-8.3) | 99.2 | <0.001 |  |  | 0.15 |
|  |  | CG | 3 | 17,932 | 550 | 6.3 (2.2-12.3) | 99.4 | <0.001 |  |  | 0.14 |
| **Age, median 38.5 years** |  |  |  |  |  |  |  |  |  |  |  |
|  | Overall |  |  |  |  |  |  |  | 0.003 |  |  |
|  |  | MDRD | 44 | 166,157 | 9,234 | 6.4 (5.2-7.7) | 98.9 | <0.001 |  | 0.54 | 0.16 |
|  |  | CKD-EPI | 14 | 41,791 | 1,398 | 4.8 (2.9-7.1) | 98.7 | <0.001 |  | 0.98 | 0.15 |
|  |  | CG | 19 | 59,414 | 5,308 | 12.3 (8.4-16.7) | 99.5 | <0.001 |  | 0.17 | 0.15 |
|  | Above median |  |  |  |  |  |  |  | 0.33 |  |  |
|  |  | MDRD | 27 | 78,099 | 5,799 | 6.1 (4.6-7.9) | 98.7 | <0.001 |  |  | 0.66 |
|  |  | CKD-EPI | 6 | 4,395 | 200 | 4.8 (2.3-8.2) | 91.8 | <0.001 |  |  | 0.52 |
|  |  | CG | 6 | 43,844 | 2,906 | 8.5 (4.5-13.6) | 99.4 | <0.001 |  |  | 0.69 |
|  | Below Median |  |  |  |  |  |  |  | 0.03 |  |  |
|  |  | MDRD | 17 | 87,190 | 3,455 | 6.9 (5.2-8.8) | 98.7 | <0.001 |  |  | 0.02 |
|  |  | CKD-EPI | 8 | 37,396 | 1,198 | 4.8 (2.6-7.5) | 99.2 | <0.001 |  |  | 0.26 |
|  |  | CG | 13 | 15,570 | 2,402 | 14.2 (7.9-21.9) | 99.3 | <0.001 |  |  | 0.95 |
| **Hypertension** |  |  |  |  |  |  |  |  |  |  |  |
|  | HIV/No HTN |  |  |  |  |  |  |  | - |  |  |
|  |  | MDRD | 10 | 22,649 | 870 | 5.4 (3.4-7.9) | 97.9 | <0.001 | - | <0.001 | 0.15 |
|  |  | CKD-EPI | 1 | 3,746 | 198 | 5.3 (4.6-6.0) | - | - |  |  | - |
|  |  | CG | - | - | - | - | - | - | - | - | - |
|  | HIV/HTN |  |  |  |  |  |  |  |  |  |  |
|  |  | MDRD | 10 | 7,012 | 1,118 | 20.7 (14.3-27.8) | 95.6 | <0.001 | - | - | 0.29 |
|  |  | CKD-EPI | 1 | 891 | 88 | 9.9 (8.1-12.0) | - | - |  |  | - |
|  |  | CG | - | - | - | - | - | - | - | - | - |
| **Hypertension prevalence (median 19%)** |  |  |  |  |  |  |  |  |  |  |  |
|  | Overall |  |  |  |  |  |  |  | 0.08 |  |  |
|  |  | MDRD | 23 | 55,920 | 4,897 | 5.3 (3.8-7.0) | 98.2 | <0.001 |  | 0.30 | 0.02 |
|  |  | CKD-EPI | 9 | 17,564 | 453 | 3.3 (1.8-5.2) | 96.8 | <0.001 |  | 0.42 | 0.19 |
|  |  | CG | 6 | 19,289 | 826 | 10.4 (3.5-20.4) | 99.1 | <0.001 |  | <0.001 | 0.10 |
|  | Above median |  |  |  |  |  |  |  | <0.001 |  |  |
|  |  | MDRD | 12 | 21,638 | 1,495 | 6.2 (4.2-8.5) | 96.7 | <0.001 |  |  | 0.60 |
|  |  | CKD-EPI | 6 | 8,870 | 216 | 3.9 (1.3-7.6) | 97.9 | <0.001 |  |  | 0.11 |
|  |  | CG | 1 | 723 | 47 | 6.5 (4.9-8.5) | - | - |  |  | - |
|  | Below median |  |  |  |  |  |  |  | 0.05 |  |  |
|  |  | MDRD | 11 | 34,941 | 3,402 | 4.3 (2.2-7.2) | 98.7 | <0.001 |  |  | 0.04 |
|  |  | CKD-EPI | 3 | 8,694 | 237 | 2.7 (2.4-3.0) | 0.0 | <0.001 |  |  | 0.09 |
|  |  | CG | 5 | 18,566 | 779 | 11.3 (2.6-24.8) | 99.1 | <0.001 |  |  | 0.14 |
| **Diabetes** |  |  |  |  |  |  |  |  |  |  |  |
|  | HIV/ No DM |  |  |  |  |  |  |  |  |  |  |
|  |  | MDRD | 10 | 22,620 | 1644 | 8.4 (5.5-11.8) | 98.2 | <0.001 | - | <0.001 | 0.38 |
|  |  | CKD-EPI | 2 | 11,702 | 456 | 3.7 (3.4-4.1) | 99.1 | <0.001 |  |  | - |
|  |  | CG | - | - | - | - | - | - |  |  | - |
|  | HIV/DM |  |  |  |  |  |  |  |  |  |  |
|  |  | MDRD | 10 | 2403 | 339 | 19.4 (13.5-26.0) | 83.6 | <0.001 | - | - | 0.08 |
|  |  | CKD-EPI | 2 | 320 | 36 | 11.2 (8.0-15.0) | 99.1 | <0.001 |  |  | - |
|  |  | CG | - | - | - | - | - | - |  |  | - |
| **Diabetes prevalence (median 6.1%)** |  |  |  |  |  |  |  |  |  |  |  |
|  | Overall |  |  |  |  |  |  |  | 0.06 |  |  |
|  |  | MDRD | 23 | 60,538 | 5,172 | 5.5 (4.0-7.1) | 98.2 | <0.001 |  | 0.19 | 0.02 |
|  |  | CKD-EPI | 9 | 17,564 | 453 | 3.3 (1.8-5.2) | 96.8 | <0.001 |  | 0.06 |  |
|  |  | CG | 6 | 19,760 | 870 | 10.4 (3.8-19.7) | 99.1 | <0.001 |  | <0.001 |  |
|  | Above median |  |  |  |  |  |  |  | <0.001 |  |  |
|  |  | MDRD | 14 | 48,857 | 4,618 | 6.1 (4.3-8.3) | 98.4 | <0.001 |  |  | 0.06 |
|  |  | CKD-EPI | 4 | 3,263 | 165 | 5.6 (2.3-10.2) | 94.5 | <0.001 |  |  | 0.53 |
|  |  | CG | 1 | 723 | 47 | 6.5 (4.9-8.5) | - | - |  |  | - |
|  | Below median |  |  |  |  |  |  |  | 0.02 |  |  |
|  |  | MDRD | 9 | 11,681 | 554 | 4.4 (3.0-6.2) | 91.3 | <0.001 |  |  | 0.84 |
|  |  | CKD-EPI | 5 | 14,301 | 288 | 2.0 (0.8-3.8) | 96.7 | <0.001 |  |  | 0.90 |
|  |  | CG | 5 | 19,037 | 823 | 11.2 (3.1-23.4) | 99.3 | <0.001 |  |  | 0.11 |
| **Hepatitis B** |  |  |  |  |  |  |  |  |  |  |  |
|  | HIV/ No HBV |  |  |  |  |  |  |  | <0.001 |  |  |
|  |  | MDRD | 13 | 82,428 | 2,747 | 7.5 (5.5-9.8) | 98.8 | <0.001 |  | 0.89 | 0.02 |
|  |  | CKD-EPI | 4 | 31,008 | 912 | 3.6 (2.0-5.6) | 98.3 | <0.001 |  |  | 0.33 |
|  |  | CG | 1 | 419 | 101 | 24.1 (20.3-28.4) |  |  |  |  | - |
|  | HIV/HBV |  |  |  |  |  |  |  | 0.004 |  |  |
|  |  | MDRD | 13 | 5,741 | 236 | 7.2 (4.4-10.5) | 90.2 | <0.001 |  |  | 0.01 |
|  |  | CKD-EPI | 4 | 1,574 | 30 | 2.3 (0.8-4.3) | 64.5 | 0.04 |  | 0.44 | 0.21 |
|  |  | CG | 1 | 72 | 16 | 22.2 (14.2-33.1) | - | - |  |  | - |
| **Hepatitis B co-infection prevalence, median 5.6%** |  |  |  |  |  |  |  |  |  |  |  |
|  | Overall |  |  |  |  |  |  |  | 0.03 |  |  |
|  |  | MDRD | 18 | 66,090 | 4,376 | 4.3 (2.5-6.5) | 99.2 | <0.001 |  | 0.98 | 0.33 |
|  |  | CKD-EPI | 6 | 34,969 | 728 | 2.0 (1.1-3.1) | 96.4 | <0.001 |  | 0.81 | 0.88 |
|  |  | CG | 4 | 18,295 | 636 | 8.1 (1.9-17.9) | 98.8 | <0.001 |  | <0.001 | 0.29 |
|  | Above median |  |  |  |  |  |  |  | 0.03 |  |  |
|  |  | MDRD | 10 | 17,010 | 883 | 4.3 (3.1-5.6) | 92.3 | <0.001 |  |  | 0.49 |
|  |  | CKD-EPI | 2 | 845 | 18 | 2.1 (1.2-3.2) | 99.3 | <0.001 |  |  | - |
|  |  | CG | 2 | 17,327 | 577 | 3.0 (2.8-3.3) | 99.6 | <0.001 |  |  | - |
|  | Below median |  |  |  |  |  |  |  | <0.001 |  |  |
|  |  | MDRD | 8 | 49,080 | 3,493 | 4.3 (1.3-8.9) | 99.7 | <0.001 |  |  | 0.64 |
|  |  | CKD-EPI | 4 | 34,124 | 710 | 2.0 (1.0-3.4) | 97.8 | <0.001 |  |  | 0.84 |
|  |  | CG | 2 | 968 | 59 | 6.0 (4.6-7.7) | 99.6 | <0.001 |  |  | - |
| **Hepatitis C** |  |  |  |  |  |  |  |  |  |  |  |
|  | HIV/No HCV |  |  |  |  |  |  |  | <0.001 |  |  |
|  |  | MDRD | 14 | 71,799 | 2,695 | 7.3 (5.6-9.3) | 98.3 | <0.001 |  | 0.74 | 0.01 |
|  |  | CKD-EPI | 4 | 29,286 | 842 | 3.5 (1.9-5.5) | 98.2 | <0.001 |  |  | 0.36 |
|  |  | CG | 1 | 433 | 101 | 23.3 (19.6-27.5) | - |  |  |  |  |
|  | HIV/HCV |  |  |  |  |  |  |  | 0.06 |  |  |
|  |  | MDRD | 14 | 11,183 | 598 | 7.6 (4.5-11.4) | 97.1 | <0.001 |  |  | 0.10 |
|  |  | CKD-EPI | 4 | 3,649 | 93 | 3.7 (1.8-6.2) | 87.3 | <0.001 |  | 0.78 | 0.08 |
|  |  | CG | 1 | 58 | 16 | 27.6 (17.8-40.2) | - |  |  |  |  |

| **Hepatitis C co-infection prevalence, median – 11%** |  |  |  |  |  |  |  |  |  |  |  |
| --- | --- | --- | --- | --- | --- | --- | --- | --- | --- | --- | --- |
|  |  |  |  |  |  |  |  |  | 0.03 |  |  |
|  | Overall | MDRD | 21 | 109,968 | 5,914 | 4.5 (3.0-6.2) | 99.2 | <0.001 |  | 0.18 | 0.96 |
|  |  | CKD-EPI | 6 | 35,102 | 752 | 2.3 (1.4-3.6) | 96.9 | <0.001 |  | 0.72 | 0.56 |
|  |  | CG | 4 | 18,295 | 636 | 8.1 (1,9-17.9) | 98.8 | <0.001 |  |  | 0.29 |
|  | Above median |  |  |  |  |  |  |  | <0.001 |  |  |
|  |  | MDRD | 12 | 82,459 | 5,192 | 5.3 (3.1-8.1) | 99.4 | <0.001 |  |  | 0.96 |
|  |  | CKD-EPI | 2 | 7,580 | 209 | 2.6 (2.3-3.0) | 99.3 | <0.001 |  |  | - |
|  |  | CG | 2 | 1,214 | 164 | 12.4 (10.6-14.3) | 99.6 | <0.001 |  |  | - |
|  | Below median |  |  |  |  |  |  |  | 0.39 |  |  |
|  |  | MDRD | 9 | 27,509 | 722 | 3.5 (2.3-4.8) | 93.2 | <0.001 |  |  | 0.16 |
|  |  | CKD-EPI | 4 | 27,522 | 543 | 2.4 (1.0-4.3) | 97.8 | <0.001 |  |  | 0.58 |
|  |  | CG | 2 | 17,081 | 472 | 2.7 (2.4-2.9) | 99.6 | <0.001 |  |  | - |

*HTN=Hypertension; DM=Diabetes Mellitus; HBV=Hepatitis B; HCV=Hepatitis C*
