# Supplementary figures and images for: Chronic kidney disease in the global adult HIV-infected population: A systematic review and meta-analysis
Source: PLoS One. 2018 Apr 16;13(4):e0195443. doi: 10.1371/journal.pone.0195443 (PMC5901989; doi:10.1371/journal.pone.0195443)

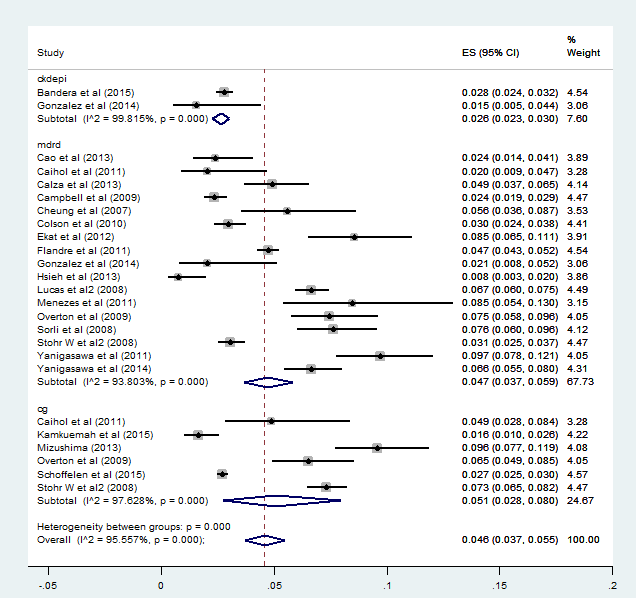

Supplement: S1 Fig — (TIF) [file pone.0195443.s006.tif]

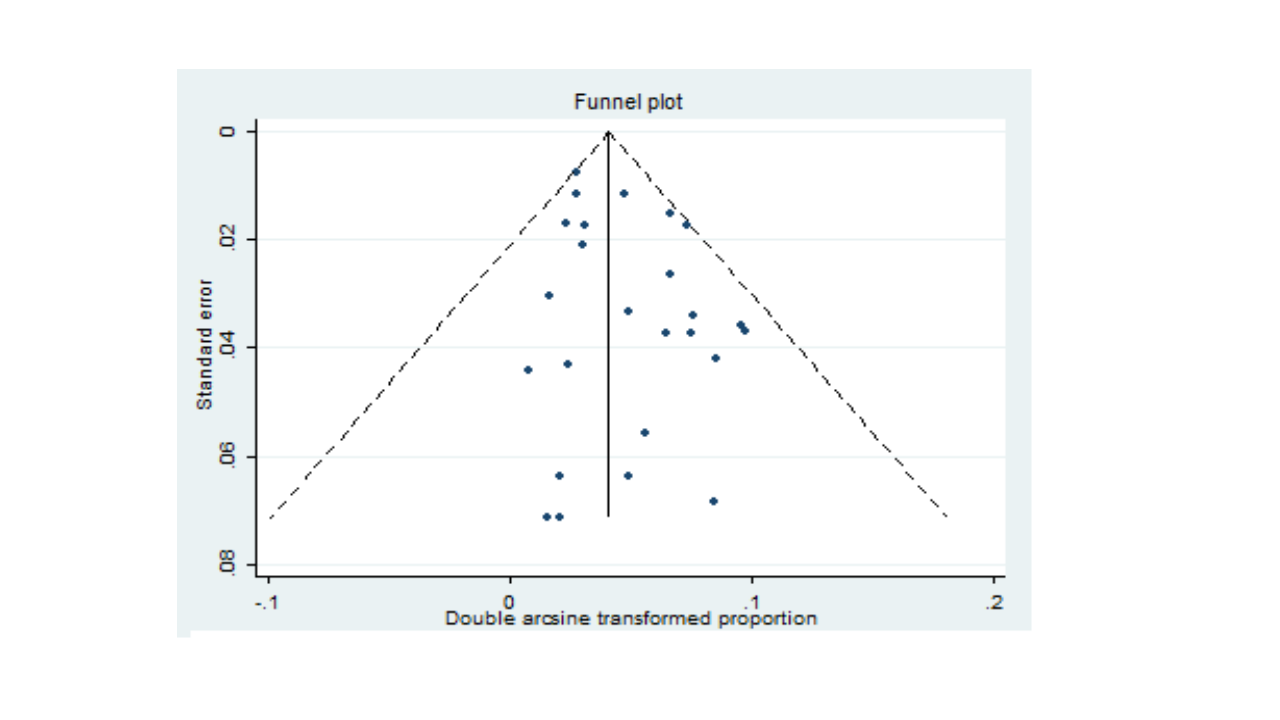

Supplement: S2 Fig — (TIF) [file pone.0195443.s007.tif]

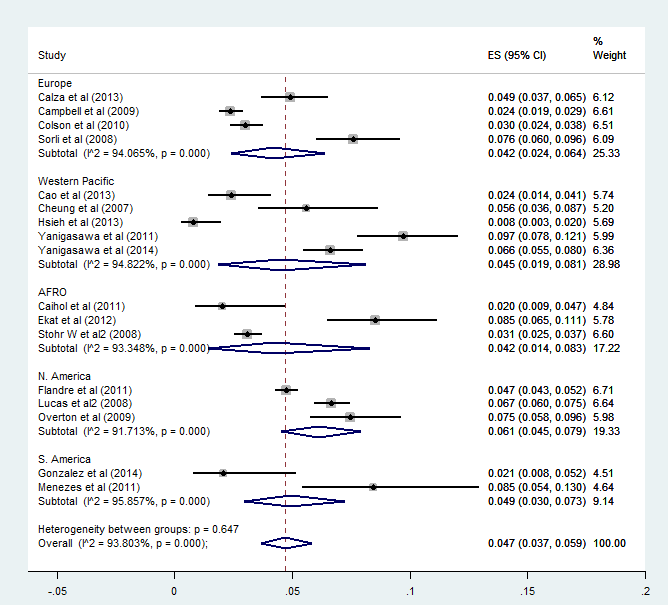

Supplement: S3 Fig — (TIF) [file pone.0195443.s008.tif]
